# Determination of Pyrethroids in* Paris polyphylla* Sample by High-Performance Liquid Chromatography Using Ultrasound-Assisted Magnetic Solid-Phase Extraction

**DOI:** 10.1155/2019/4968924

**Published:** 2019-04-24

**Authors:** Wei Zhang, Xin Zhao, Hengling Meng, Jin Yang, Xiang Wei, Yong Min

**Affiliations:** ^1^Honghe University, Mengzi, China; ^2^Key Laboratory of Ethnomedicine (Minzu University of China), Ministry of Education, Beijing 100081, China

## Abstract

A novel ultrasound-assisted magnetic solid-phase extraction (UA-MSPE) was developed for the separation/preconcentration of trace amounts of pyrethroids (fenpropathrin, fenvalerate, deltamethrin, and bifenthrin) in* Paris polyphylla* sample using carbon nanotubes based on Fe_3_O_4_ magnetic nanoparticles (Fe_3_O_4_@CNT MNPs), and high-performance liquid chromatography-UV is described. High recoveries of pyrethroids were obtained at a low MNPs concentration because sonication enhances the contact chances between magnetic nanoparticles and extractable analytes and promotes the extractability of the MSPE process. After the extraction, the adsorbent can be conveniently separated from the sample solution by an external magnet, and the adsorbed analytes were eluted from magnetic Fe_3_O_4_@CNT. The main factors influencing the extraction efficiency including the amount of the MNPs, the extraction time, the pH of sample solution, the sonicating time, and the desorption conditions were studied and optimized. Under the optimized experimental conditions, a good linearity was observed in the range of 1-100.0 ng mL^−1^ for all the analytes, with the correlation coefficients (r) ranging from 0.9962 to 0.9991. The limits of detection of the four pyrethroids are 0.53, 0.26, 0.47, and 0.67 ng mL^−1^, respectively. The recoveries of the method were in the range between 85.5% and 93.2%. This method is much faster and more effective than traditional SPE methods, and it is promising for the analysis of pyrethroids residues.

## 1. Introduction

*Paris polyphylla* is a famous traditional Chinese medicinal herb with anti-inflammatory and hematischesis properties and has been shown recently to possess anticancer activity [1]. However,* Paris polyphylla* is easily attacked by several pests and diseases. Some pyrethroid pesticides, such as fenpropathrin, fenvalerate, deltamethrin, and bifenthrin, have been widely applied to control and prevent mites, leafhoppers, plant bugs, and aphids in* Paris polyphylla* because of their excellent insecticidal activity, fast knockdown capability, and relatively low mammalian toxicity [2, 3] and have been gradually taking the place of organophosphorus and carbamate pesticides. However, due to bioaccumulation through the food chain, they can eventually become a risk or threat to both animal and human life [4].

Numerous determination methods, such as gas chromatography-electron capture detection [5], gas chromatography-mass spectrometry [6–8], high-performance liquid chromatography-UV detection [9, 10], and high-performance liquid chromatography-mass spectrometry [11], have been reported to determine pyrethroid residues. Meanwhile, in the analysis of real samples, the presence of potential contaminants, the complexity of matrixes, the ultratrace concentration of analytes, and the need for achieving increasingly lower detection limits make the use of miniaturized or automated sample pretreatment techniques necessary prior to their determination, as has been demonstrated in several recent review articles or monographs. Sample pretreatment is an important step in a chemical analysis, especially in the analysis of pesticide residues in medicinal herbs samples. Generally, liquid-liquid extraction (LLE) [12] and solid-phase extraction (SPE) [13] are the most widely used techniques for the preconcentration and separation of the compounds from medicinal herb samples. However, LLE is not preferred when water either containing emulsifying agents or analytes is present in trace quantities. In addition, it is time consuming and requires large volume of toxic organic solvents. SPE plays key role in obtaining higher enrichment efficiency of analytes and requires much less amount of organic solvents than LLE, but SPE can still be tedious, time consuming, and relatively expensive.

Magnetic solid-phase extraction (MSPE) based on functionalized magnetic materials has received considerable attention in recent years, especially as a promising sample preparation technique [14]. MSPE with magnetic nanoparticles (MNPs) as the adsorbents can offer several advantages over traditional LLE and SPE, such as having very high surface areas and a short diffusion route, which results in high extraction capacity and efficiency. In this study, magnetic carbon nanotubes (Mag-CNTs) have been synthesized by chemical deposition of Fe_3_O_4_ nanoparticles onto carbon nanotubes combining the high adsorption capacity of CNTs with the convenient separation of Fe_3_O_4_ in one material [15]. MNPs are added to the sample solution and the target analyte is adsorbed on the surface of the magnetic sorbents under stirring. The sorbents are separated from the suspension solution by means of an external magnetic force. Then, the target analytes are desorbed by the eluent for further determination. Compared with traditional adsorbents, Mag-CNTs can make separation process easier and faster without the need for additional centrifugation or filtration procedures and also can avoid the time-consuming column passing operations encountered in traditional SPE.

In this work, Mag-CNTs were synthesized by the in situ chemical coprecipitation of Fe^2+^ and Fe^3+^ in an alkaline solution in the presence of CNT and their application for the enrichment of some pyrethroids from* Paris polyphylla* samples, followed by HPLC-UV detection. Under the optimal conditions, good recoveries and precisions were obtained.

## 2. Experimental

### 2.1. Reagents and Solutions

All chemicals were of analytical grade and prepared with double distilled water. FeCl_3_*∙*6H_2_O (99% w/w), ammonium nitrate (99% w/w), FeCl_2_*∙*4H_2_O (99% w/w), carbon nanotubes of diameter 20-30 nm and length ~50 *μ*m, standards of fenpropathrin, fenvalerate, deltamethrin, and bifenthrin were purchased from Aladdin Chemical Co. Ltd. (Shanghai, China). Acetonitrile of HPLC grade was purchased from Merck (Darmstadt, Germany). Stock standard solutions of pyrethroids (1000 ng mL^−1^) and working standard solutions were prepared in acetonitrile and stored at 4°C in brown glass vials.

### 2.2. Instrumentation and Chromatographic Conditions

A scanning electron microscopy (SEM) system VEGA3 SBH (Tescan, Czech Republic) with a tungsten electron gun was used to provide electron beam irradiation for characterization of Fe_3_O_4_ NPs and Fe_3_O_4_@CNT. A transmission electron microscope (TEM) scanning system JEM-100CXII (Japan Electronics Co.) was used for characterization of Fe_3_O_4_@CNT nanoparticles. The pH measurements were carried out with pH-meter Sartorius PB10 (Göttingen, Germany), and vacuum drying oven BPZ-6033 (Shanghai, China) was used to dry synthesized nanomaterials. An ultrasonic bath (Shanghai, China) was used for ultrasound-assisted. Chromatographic analysis was performed using an Agilent 1260 HPLC system coupled with ultraviolet detector. Separation of four pyrethroid pesticides was carried out using an Agilent C18 column (250 mm × 4.6 mm, 5 *μ*m) at 25°C, with an isocratic elution of acetonitrile-water (80:20, v/v) for 30 min at a constant flow rate of 1 mL min^−1^. The detection wavelength was 230 nm.

## 3. Methods

### 3.1. Synthesis of Fe_*3*_O_*4*_@CNT

The magnetic nanoparticles were synthesized based on previous reports using some modifications [16, 17]. 4 g of carbon nanotubes was suspended in 200 mL of mixed solution containing FeCl_3_·6H_2_O (17.4 mmol) and FeCl_2_·4H_2_O (8.7 mmol), under ultrasonication for about 10 min. Then the sodium acetate (30 g) was added and dissolved, followed by adding 100 mL ethanediamine with stirring for 20 min. The homogenous black solution obtained was transferred to a Teflon-lined stainless-steel autoclave and sealed to heat at 200°C. The reaction was allowed to be continued for 8 h. The suspension was cooled to room temperature, and Fe_3_O_4_@CNT was isolated from the mixture with the help of a permanent magnet. Separated Fe_3_O_4_@CNT was washed three times with deionized water followed by ethanol to remove unreacted reagents. The obtained Fe_3_O_4_@CNT NPs were redispersed in 50 mL of deionized water via sonication and the concentration of Fe_3_O_4_@CNT NPs suspension was estimated to be about 120 mg mL^−1^.

### 3.2. Sample Preparation and MSPE Procedure

**Figure 1:**
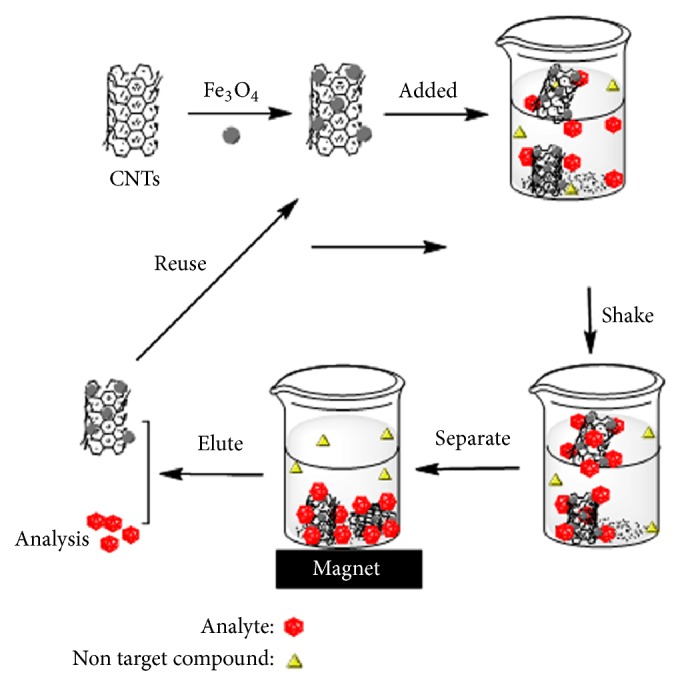
Schematic illustration of extraction procedure.

2.0 g of crushed* Paris polyphylla*, accurately measured into a 100 ml conical flask, was added to 20 mL of n-hexane and placed on a shaker for 20 min, and then the mixture was centrifuged for 5 min. The supernatant was transferred to a 50 mL beaker, and 500 *μ*L of Fe_3_O_4_@CNT suspension was added by a pipette. The mixture solution was under ultrasonic treatment at room temperature for 2 min. The magnetic adsorbents with absorbed pyrethroids were isolated rapidly from the solution by a strong magnet clinging to the outer wall of the beaker, and the supernatant had been poured away, as shown schematically in Figure 1. Subsequently, the preconcentrated pyrethroids were eluted from the magnetic nanoparticles with 2 mL 90% acidified acetonitrile for further liquid chromatography analysis. The blank tests were carried out under the same conditions with blank solution without adding any analytes.

## 4. Results and Discussion

### 4.1. Characterization of Composite

**Figure 2:**
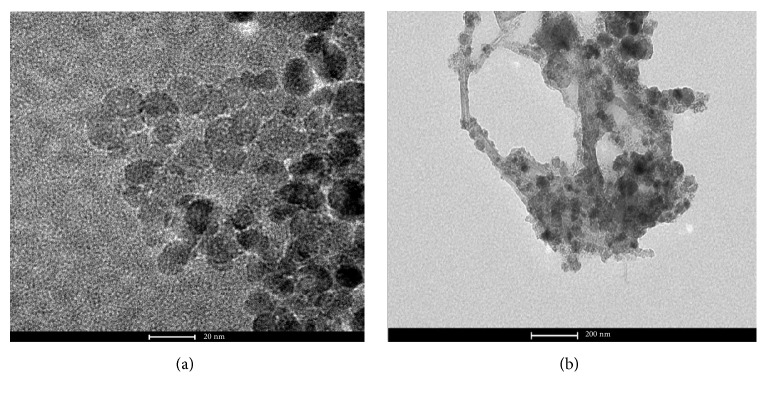
The SEM images of Fe_3_O_4_ MNPs (a), the TEM image of Fe_3_O_4_@CNTs MNPs-diatomite composite (b).

**Figure 3:**
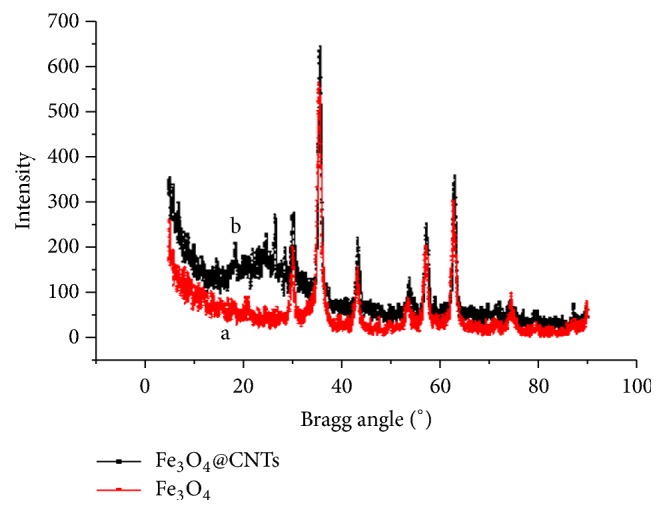
X-ray diffraction patterns of (a) Fe_3_O_4_ MNPs and (b) Fe_3_O_4_@CNTs MNPs.

The SEM images of synthesized Fe_3_O_4_ NPs (Figure 2(a)) showed homogeneous distribution of particles, and diameters of nanomaterials synthesized were in the range of 10-50 nm. Transmission electron micrographs (TEM) of the synthesized magnetic carbon nanotubes (Figure 2(b)) showed that iron oxide nanoparticles were successfully attached onto the surface of carbon nanotubes. The results are in good agreement with reports in the literature [6]. The XRD analysis (Figure 3) was applied to investigate the crystalline structures of the magnetic nanoparticles; diffraction peaks with 2*θ* of 31.2°, 36.1°, 44.5°, 55.8°, 58.5°, and 63.6° were observed, indicating a cubic spinel structure of the Fe_3_O_4_ magnetite. And the Fe_3_O_4_@CNT MNPs show the typical peak of carbon nanotubes at 2*θ*=26.8°. Figure 3 shows the X-ray diffraction of magnetic carbon nanotubes which includes all the carbon nanotubes and Fe_3_O_4_ peaks.

### 4.2. Optimization of the MSPE Conditions

In order to evaluate the applicability of CNTs for extraction and determination of pyrethroids in* Paris polyphylla* samples, the parameters affecting the performance of the extraction, such as sample pH, Fe_3_O_4_@CNTs amount, sonicating time, and elute solvent, were investigated. 50 mL of* Paris polyphylla* samples solution spiked with 100 ng mL^−1^ pyrethroids was used to study the optimum MSPE conditions.

### 4.3. Effect of pH

**Figure 4:**
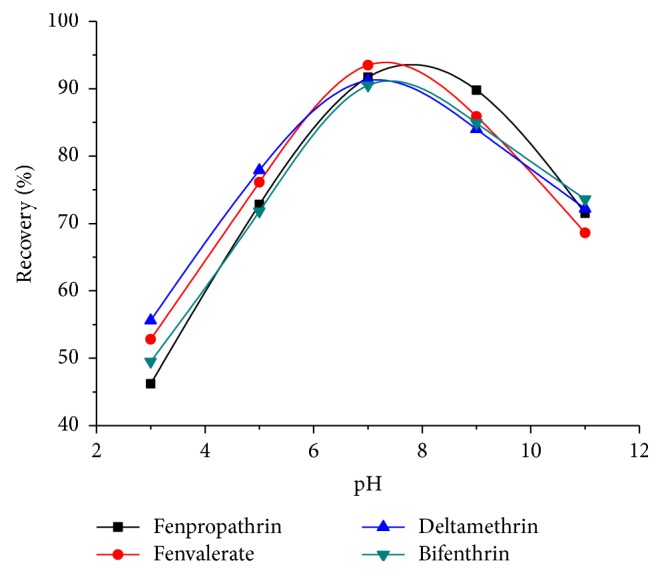
Effect of pH. extraction conditions: sample volume, 50.0 mL spiked with 100 ng mL^−1^ of four pyrethroids; magnetic nanoparticles (MNPs), 60 mg; ultrasonication time, 2 min; eluent solvent, 2 mL of acetonitrile /acetic acid (9:1, v/v).

Sample solution pH determines the form of analytes in solution and the surface charge of sorbent which plays an important role in the extraction of pyrethroids. The influence of the pH on the extraction efficiencies was investigated in order to find a pH value at which the extraction of the pyrethroids was enhanced. When varying the pH value from 3 to 11, significant effect was observed on extraction efficiency. It can be seen that poor recoveries were obtained when sample solutions were acidic. With the pH increasing, the recoveries of pyrethroids increased and reached plateau values with pH close to 7.0 and then decreased for all pyrethroids when the pH values were higher than 7, as shown in Figure 4. Finally, a sample pH of 7.0 was selected to guarantee excellent adsorption rate for subsequent work.

### 4.4. Effect of Magnetic Nanoparticles Amount

**Figure 5:**
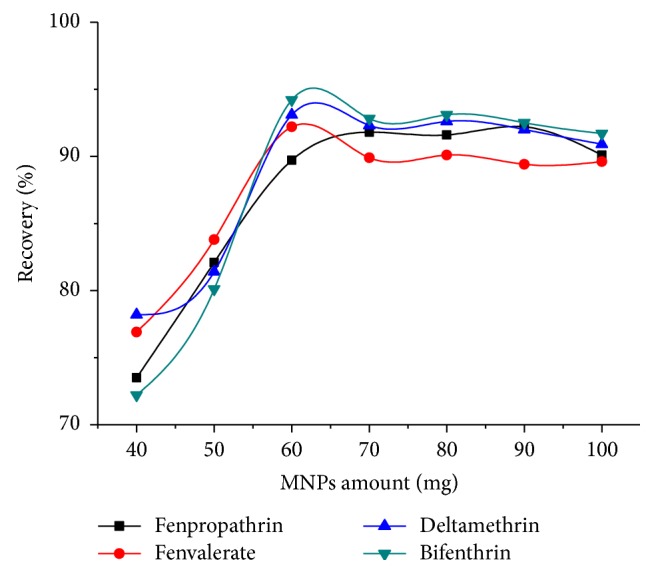
Effect of the amount of MNPs. Extraction conditions: sample volume, 50.0 mL spiked with 100 ng mL^−1^ of four pyrethroids; pH, 7.0; ultrasonication time, 2 min; eluent solvent, 2 mL of acetonitrile/acetic acid (9:1, v/v).

The effect of MNPs amount on the extraction efficiency of the analytes was investigated by varying the sorbent within the range of 40-100 mg. The results, as illustrated in Figure 5, show that the recovery of pyrethroids increases with the increase in MNPs amount up to 60 mg, and then it remains constant. High surface area of nanosized sorbents can explain the low amount usage of the nanosized sorbents compared to conventional SPE sorbents. In addition, CNT coated MNPs present both the high adsorption capacity of carbon nanotubes and the convenient separation of Fe_3_O_4_ MNPs, providing a dual mechanism for extraction of the analyte, and decrease the required adsorbent amount. Thus, the optimization process was performed taking into consideration Fe_3_O_4_@CNT MNPs amount at 60 mg.

### 4.5. Effect of Sonication

**Figure 6:**
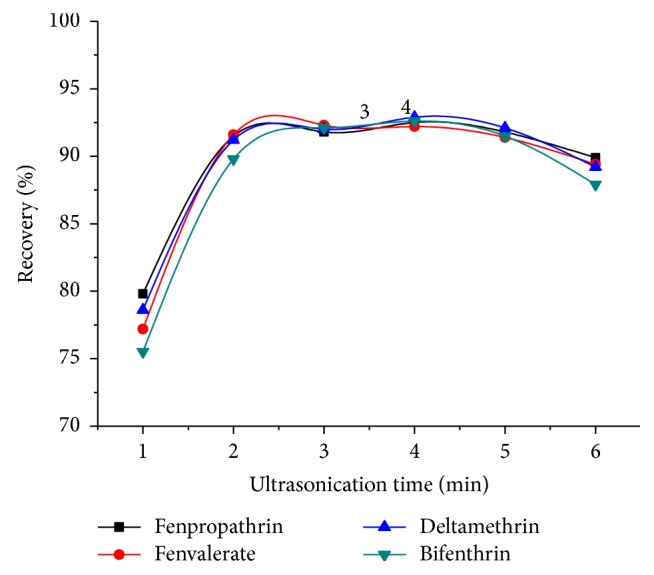
Effect of ultrasonication time. Extraction conditions: sample volume, 50.0 mL spiked with 100 ng mL^−1^ of four pyrethroids; pH, 7.0; magnetic nanoparticles (MNPs), 60 mg; eluent solvent, 2 mL of acetonitrile/acetic acid (9:1, v/v).

In the process of adsorption, the contact time is one of the prime factors affecting the target analytes extraction. When the MNPs were separated immediately without a contact process into the sample, the recovery of the four analytes was below 50%. Ultrasound can accelerate the interactive rate between the MNPs and solution so that the target analytes could be well adsorbed on the surface of MNPs in a shorter time. The effect of ultrasonication time in the range of 0-6 min was evaluated at 25 Hz of ultrasonication frequency and 25°C water bath temperature to reveal the effect of extraction time on the recovery of the pyrethroids. As indicated in Figure 6, the extraction recovery of the pyrethroids reached a maximum at 2 min. Therefore, 2 min of ultrasound time was chosen for the following experiments.

### 4.6. Effect of Desorption Conditions

Since the* Paris polyphylla* samples contain complex matrix components, a washing step is required to remove the interfering compounds from sample matrixes without desorbing the target analytes. Different percentages of methanol aqueous solutions were used as the washing solutions. There was little change in the pyrethroids recoveries after washing when the percentage of methanol in the washing solutions was in the range of 1%-5%. The recoveries of pyrethroids decreased with further increased methanol in the washing solution. In this study, 5 mL 2% aqueous methanol was used as washing solution.

**Figure 7:**
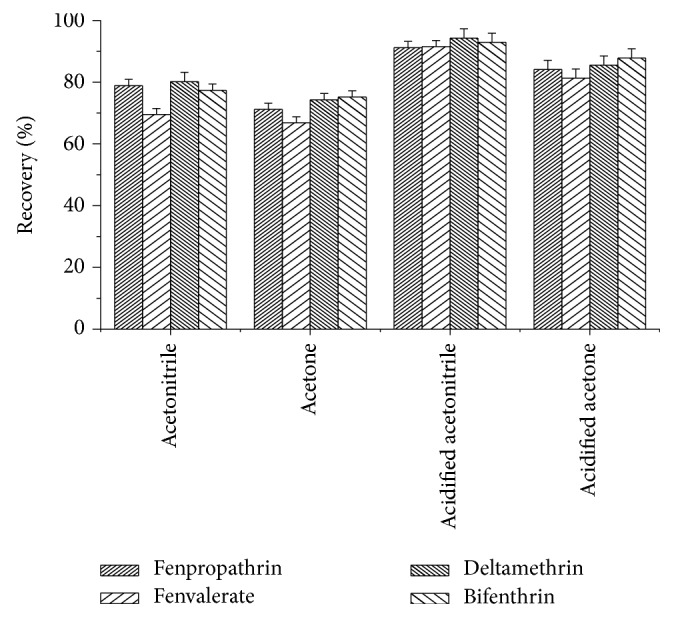
Effect of desorption solution. Extraction conditions: sample volume, 50.0 mL spiked with 100 ng mL^−1^ of four pyrethroids; pH, 7.0; ultrasonication time, 2 min; magnetic nanoparticles (MNPs), 60 mg.

Once the Fe_3_O_4_@CNT MNPs and adsorbed analytes are separated from the washing solution again, an elution step with an organic solvent is required to remove the analytes from the nanoparticle. Different organic solvents (acetonitrile, acetone, acidified acetonitrile, and acidified acetone) (Figure 7) and the effect of desorption solution volume on desorption efficiency of the analytes were also investigated. Unsatisfactory recoveries were found by using acetonitrile and acetone. The best recoveries were obtained using 2 mL mixture of acetonitrile/acetic acid (9:1, v/v) as eluting solution.

### 4.7. Effect of Other Molecules

The investigation of matrix effects validated the selectivity of the procedure for pyrethroids adsorption due to the competition of other residues of pesticide for available adsorption on the nanoparticles. In this work, the tolerance limit was defined as the amount of foreign molecules such as benzex, dicophane, and dimethoate causing a change in the peak area of less than ±5%. The effect of some common molecules coexisting in* Paris polyphylla* sample on the adsorption of 500.0 ng mL^−1^, respectively, with 60 mg Fe_3_O_4_@CNT MNPs was investigated. Most of the investigated species did not interfere even when presenting 100-200-fold excess of pyrethroids. The results confirm good selectivity of the proposed method and applicability of the method to the accurate determination of pyrethroids in real samples.

### 4.8. Validation

The limits of detection (LODs), repeatability (RSDs), linearity, and correlation coefficients (R^2^) were studied. The analytical figures of merit, obtained under the MSPE optimum conditions, are summarized in Table 1. Calibration curves were obtained by plotting the peak area of the studied analytes versus the theoretical concentration of the analytes added to* Paris polyphylla* samples. All analytes exhibited a linear range from 1 to 100 ng mL^−1^, with R^2^ varying between 0.9962 and 0.9991. The LODs, calculated as a concentration at a signal-to-noise ratio of 3, were estimated to be about 0.53 ng mL^−1^ for fenpropathrin, 0.26 for fenvalerate, 0.47 ng mL^−1^ for bifenthrin, and 0.67 ng mL^−1^ for deltamethrin, respectively, as shown in Table 1. The repeatability, described as relative standard deviations (RSDs) of five replicate measurements at concentration level of 100 ng mL^−1^, was in the range of 3.67-5.32%. A comparison of the method with other preconcentration techniques for pyrethroids is presented in Table 3. The results show that the adsorbent has comparable recoveries for pyrethroids as the previous methods. The method is rapid, inexpensive, and highly sensitive to the determination of pyrethroids and was employed for the analysis of* Paris polyphylla*. In addition, the proposed method uses less adsorbent and less organic solvent than the others.

### 4.9. Matrix Effects

A series of experiments of interference substances have been carried out to evaluate the impact of matrix effects. Several coexisting ions, such as Na^+^, K^+^, Mg^2+^, Cl^−^, SO_4_^2−^, and ascorbic acid, that may exist in* Paris polyphylla* samples were examined. The results indicate that larger quantities of the coexisting ions and organics do not interfere with the determination. They show that the proposed method was suitable for the analysis of* Paris polyphylla* samples with complex matrix.

### 4.10. Application to Analysis of Real Samples

The developed method was utilized for the quantification of pyrethroids in commercial* Paris polyphylla* by spiking with the standards of the pyrethroid at three concentrations of 20, 50, and 100 ng g^−1^.  Table 2 shows the results as the mean value of three independent measurements, together with standard deviations. Measured concentrations of fenpropathrin (FP), fenvalerate (FV), deltamethrin (DT), and bifenthrin (BT) in samples ranged between 17.1 and 101.5 ng g^−1^. Recoveries obtained ranged between 85.5% and 101.5% with relative standard deviations from 3.7% to 5.6%. The obtained results in Table 2 show that the proposed method is suitable for pyrethroid analysis in real* Paris polyphylla* samples.

**Figure 8:**
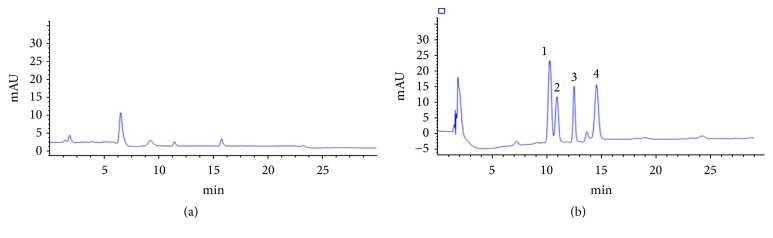
HPLC chromatograms of (a) the blank* Paris polyphylla* sample after MSPE (b) and the* Paris polyphylla* sample spiked with 50 ng g^−1^ of each pyrethroid, (1) fenpropathrin, (2) fenvalerate, (3) deltamethrin, (4) bifenthrin, after MSPE.

Figure 8 shows representative chromatograms of the extracted analytes from* Paris polyphylla* samples spiked with each of the pyrethroids at 50 ng g^−1^. Chromatographic peaks in samples indicate the absence of different pyrethroids. Chromatograms are clear enough for the detection and quantitation of pyrethroids in the samples analysis.

## 5. Conclusion

In this work, CNT-based magnetic nanoparticles were synthesized and successfully applied for the efficient separation and preconcentration of trace pyrethroids from* Paris polyphylla* samples prior to analysis by HPLC-UV. The magnetic separation greatly improved the phase separation while avoiding the time-consuming column passing operations encountered in SPE. Since the use of MNPs sorbent offers high extraction capacity, rapid extraction dynamics, and high extraction efficiencies, low amount of sorbents can be used and short equilibrium time is required to extract the analytes from samples. The results showed sensitive, low detection limit, and good relative extraction recovery of the method is acceptable. Thus, the method is useful for the quality control of pyrethroids in medical herb preparations.

## Figures and Tables

**Table 1:** Analytical features of the proposed method.

Compounds	Regression equation	Linear range (ng mL^−1^)	RSD(%) n=6	r	Limit of detection (ng mL^−1^)
Fenpropathrin	Y=254.3X+2.78	1-100	4.36	0.9980	0.53
Fenvalerate	Y=176.6X+2.11	1-100	3.67	0.9991	0.26
Deltamethrin	Y=196.8X+3.78	1-100	5.32	0.9990	0.67
Bifenthrin	Y=189.5X+4.21	1-100	4.98	0.9962	0.47

**Table 2:** Mean concentrations (ng g^−1^) of pyrethroids found in *Paris polyphylla* sample of Kunming, and recoveries (%) obtained after spiking the samples with FP, FV, DT, and BT at three concentration levels.

Sample	Spiked (ng g^−1^)				Found (ng g^−1^)				Recovery (%) ± SD^a^			
	FP	FV	DT	BT	FP	FV	DT	BT	FP	FV	DT	BT
*Paris polyphylla* of Kunming	0				ND^a^	ND^a^	ND^a^	ND^a^	-	-	-	-
	20				18.5	18.1	17.9	17.1	92.5±4.6	90.5±5.1	89.5±3.7	85.5±4.8
	50				46.7	46.2	45.9	45.8	93.4±3.9	92.4±4.8	91.8±4. 5	91.6±5.6
	100				101.5	95.5	92.7	95.9	101.5±4.1	95.5±4.5	92.7±5.2	95.9±4.1

^a^Standard deviation (n = 3).

**Table 3:** Comparison of the proposed method and some other methods for pyrethroids determination.

Method	Detection	LOD (ng mL^−1^)	Recovery (%)	Reference
DLLME/D-*μ*-SPE^a^	HPLC-UV	0.05-2.0	91.7-104.5	[18]
DLLME^b^	HPLC-UV	2-5	84-94	[10]
Matrix SPE^c^	GC-MS	0.005-0.06	83±3	[19]
LSE-MSPE^d^	UFLC	0.69-1.2	76-99.5	[20]
UA-MSPE	HPLC-UV	0.26-0.67	85.5-93.2	This work

^a^Dispersive liquid microextraction combined with dispersive *μ*-solid phase extraction;  ^b^dispersive liquid-liquid microextraction;  ^c^matrix solid-phase dispersion;  ^d^liquid-solid extraction coupled with magnetic solid-phase extraction.

## Data Availability

The data used to support the findings of this study are available from the corresponding author upon request.
